# Hierarchical path planning from speech instructions with spatial concept-based topometric semantic mapping

**DOI:** 10.3389/frobt.2024.1291426

**Published:** 2024-08-01

**Authors:** Akira Taniguchi, Shuya Ito, Tadahiro Taniguchi

**Affiliations:** Emergent Systems Laboratory, Ritsumeikan University, Kusatsu, Shiga, Japan

**Keywords:** control as probabilistic inference, language navigation, hierarchical path planning, probabilistic generative model, semantic map, topological map

## Abstract

Assisting individuals in their daily activities through autonomous mobile robots is a significant concern, especially for users without specialized knowledge. Specifically, the capability of a robot to navigate to destinations based on human speech instructions is crucial. Although robots can take different paths toward the same objective, the shortest path is not always the most suitable. A preferred approach would be to accommodate waypoint specifications flexibly for planning an improved alternative path even with detours. Furthermore, robots require real-time inference capabilities. In this sense, spatial representations include semantic, topological, and metric-level representations, each capturing different aspects of the environment. This study aimed to realize a hierarchical spatial representation using a topometric semantic map and path planning with speech instructions by including waypoints. Thus, we present a hierarchical path planning method called spatial concept-based topometric semantic mapping for hierarchical path planning (SpCoTMHP), which integrates place connectivity. This approach provides a novel integrated probabilistic generative model and fast approximate inferences with interactions among the hierarchy levels. A formulation based on “control as probabilistic inference” theoretically supports the proposed path planning algorithm. We conducted experiments in a home environment using the Toyota human support robot on the SIGVerse simulator and in a lab–office environment with the real robot Albert. Here, the user issues speech commands that specify the waypoint and goal, such as “Go to the bedroom via the corridor*.*” Navigation experiments were performed using speech instructions with a waypoint to demonstrate the performance improvement of the SpCoTMHP over the baseline hierarchical path planning method with heuristic path costs (HPP-I) in terms of the weighted success rate at which the robot reaches the closest target (0.590) and passes the correct waypoints. The computation time was significantly improved by 7.14 s with the SpCoTMHP than the baseline HPP-I in advanced tasks. Thus, hierarchical spatial representations provide mutually understandable instruction forms for both humans and robots, thus enabling language-based navigation.

## 1 Introduction

Autonomous robots are often tasked with linguistic interactions such as navigation for seamless integration into human environments. Navigation using the concepts and vocabulary tailored to specific locations learned from human and environmental interactions is a complex challenge for these robots (Taniguchi et al., 2016b; Taniguchi et al., 2019). Such robots are required to construct adaptive spatial structures and place semantics from multimodal observations acquired during movements within the environment (Kostavelis and Gasteratos, 2015; Garg et al., 2020). This concept is closely linked to the anchoring problem, which is concerned with the relationships between symbols and sensor observations (Coradeschi and Saffiotti, 2003; Galindo et al., 2005). Understanding the specific place or concept to which a word or phrase refers, i.e., the denotation, is therefore crucial.

The motivation for research on this topic stems from the necessity for autonomous robots to operate effectively in human environments. This requires them to understand human language and navigate complex environments accordingly. The significance of this research lies in enabling autonomous robots to interact within human environments both effectively and intuitively, thereby assisting the users. The primary issue in hierarchical path planning is the increased computational cost owing to the complexity of the model, which poses a risk to real-time responsiveness and efficiency. Additionally, the challenge with everyday natural language commands provided by the users is the existence of specific place names that are not generally known and the occurrence of different places within an environment that share the same name. Therefore, robots need to possess environment-specific knowledge. Enhancements in the navigation success rates and computational efficiency, especially for tasks involving linguistic instructions, could significantly broaden the applications of autonomous robots; these applications would extend beyond home support to include disaster rescue, medical assistance, and more.

**FIGURE 1:**
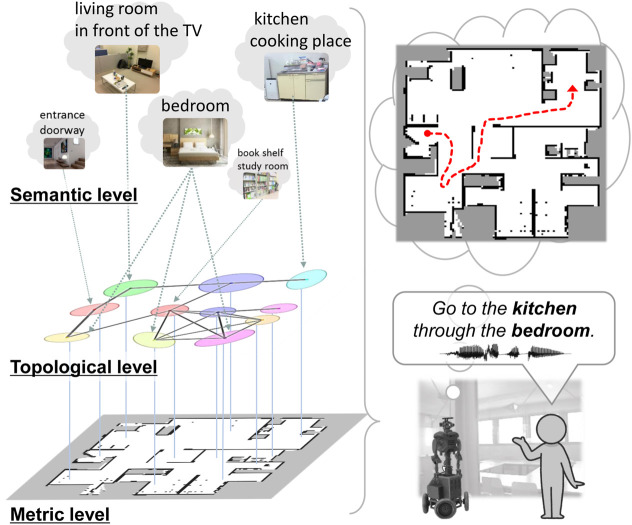
Overview of the proposed method. Left: hierarchy of spatial representation with topometric semantic mapping. Right: path planning from spoken instructions with waypoint and goal specifications.

Topometric semantic maps are a combination of metric and topological maps with semantics that are helpful for path planning using generalized place units. Thus, they facilitate human–robot linguistic interactions and assist humans. One of the key challenges here is the robot’s capacity to efficiently construct and utilize these hierarchical spatial representations for interaction tasks. Hierarchical spatial representations provide mutually understandable instruction forms for both humans and robots to enable language-based navigation. They are generalized appropriately at each level and can accommodate combinations of paths that were not considered during training. As shown in Figure 1 (left), this study entails three levels of spatial representation: (i) **semantic level** that represents place categories associated with various words and abstracted by multimodal observations; (ii) **topological level** that represents the probabilistic adjacency of places in a graph structure; (iii) **metric level** that represents the occupancy grid map and is obtained through simultaneous localization and mapping (SLAM) (Grisetti et al., 2007). In this paper, the term *spatial concepts* refers to semantic–topological knowledge grounded in real-world environments.

The main goal of this study was to realize efficient spatial representations and high-speed path planning from human speech instructions by specifying waypoints using topological semantic maps incorporating place connectivity. This study was conducted in two phases, namely spatial concept learning and path planning. **Spatial concept learning phase**: In this phase, a user guides a robot in the environment by providing natural language cues^1^, i.e., providing utterances about various locations, such as “*This is my father Bob’s study space, and it has many books*.” Furthermore, the robot collects multimodal sensor observations from the environment, including images, depth data, odometry, and speech signals. Using these sensor observations, the robot acquires knowledge of the environmental map as well as connection relationships between the places, spatial concepts, and place names. **Path planning phase**: In this phase, the robot considers speech instructions such as “*go to the kitchen*” as basic tasks and “*go to the kitchen through the bedroom*” as advanced tasks (Figure 1 (right)). In particular, this study was focused on hierarchical path planning in advanced tasks. Although the shortest paths may not always be the most suitable, robots can select alternative paths to avoid certain areas or perform specific tasks based on the user instructions. For example, the robot may choose a different route to avoid the living room with guests or to check on the pets in the bedroom. Thus, users can guide the robot to an improved path by specifying waypoints. Furthermore, when multiple locations have the same name (e.g., three bedrooms), selecting the closest route among them is appropriate. By specifying the closest waypoint to the target, the robot can accurately select the target even when many places share the same name.

In this study, “optimal” refers to the scenario that maximizes the probability of a trajectory distribution under the given conditions. Specifically, the robot should plan an overall optimal path through the designated locations. This ensures that the robot’s path planning is practical and reduces the travel distance as well as time by considering real-world constraints and objectives. It also allows greater flexibility in guiding the robot through the waypoints, thereby enabling users to direct it along preferred routes while maintaining the overall effectiveness.

This paper proposes a spatial concept-based topometric semantic mapping for hierarchical path planning (SpCoTMHP) approach with a probabilistic generative model^2^. The topometric semantic map enables path planning by combining abstract place transitions and geometrical structures in the environment. SpCoTMHP is based on a probabilistic generative model that integrates the metric, topological, and semantic levels with speech and language models into a unified framework. Learning occurs in an unsupervised manner through the joint posterior distribution derived from multimodal observations. To enhance the capture of topological structures, a learning method inspired by the function of replay in the hippocampus is introduced (Foster and Wilson, 2006). Ambiguities related to the locations and words are addressed through a probabilistic approach informed by robot experience. In addition, we develop approximate inference methods for effective path planning, where each hierarchy level influences the others. The proposed path planning is theoretically supported by the idea of *control as probabilistic inference* (CaI) (Levine, 2018), which has been shown to bridge the theoretical gap between probabilistic inference and control problems, including reinforcement learning.

The proposed approach is based on *symbol emergence in robotics* (Taniguchi et al., 2016b, 2019) and has the advantage of enabling navigation using unique spatial divisions and local names learned without annotations, which are tailored to each individual family or community environment. Hence, the users can simply communicate with the robot throughout the process from learning to task execution, thus eliminating the need for robotics expertise. Moreover, the approach is based on the robot’s real-world experiences that enable daily behavioral patterns to be captured, such as where to travel more/less frequently.

We conducted experiments in the home environment using the Toyota human support robot (HSR) on the SIGVerse simulator (Inamura and Mizuchi, 2021) and in a lab–office environment with the real robot Albert (Stachniss, 2003). SpCoTMHP was compared with baseline hierarchical path planning methods in navigation experiments using speech instructions with a designated waypoint. The main contributions of this study are as follows:

1. We demonstrated that hierarchical path planning incorporating topological maps through probabilistic inference achieves higher success rates and shorter computation times for language instructions involving waypoints compared to methods utilizing heuristic costs.
2. We illustrated that semantic mapping based on spatial concepts and considering topological maps achieves higher learning performance than SpCoSLAM, which does not incorporate topological maps.

In particular, the significance of this work is characterized by the following four items:

1. Integrated learning–planning model: The learning–planning integrated model autonomously constructs hierarchical spatial representations, including topological place connectivity, from the multimodal observations of the robot, leading to improved performances for learning and planning.
2. Probabilistic inference for real-time planning: The approximate probabilistic inference based on CaI enables real-time planning of adaptive paths from the waypoint and goal candidates.
3. Many-to-many relationships for path optimization: The probabilistic many-to-many relationships between words and locations enable planning closer paths when there are multiple target locations.
4. Spatial concepts for environment-specific planning: The spatial concepts learned in real environments are effective for path planning with environment-specific words.

The remainder of this paper is organized as follows. Section 2 presents related works on topometric semantic mapping, hierarchical path planning, and the spatial concept-based approach. Section 3 describes the proposed method SpCoTHMP. Section 4 presents experiments performed using a simulator in multiple home environments. Section 5 discusses some experiments performed in real environments. Finally, Section 6 presents the conclusions of this paper.

## 2 Related works

This section describes topometric semantic mapping in Section 2.1, hierarchical path planning in Section 2.2, robotic planning using large language models (LLMs) and foundation models in Section 2.3, and the spatial concept-based approach in Section 2.4. Table 1 displays the main characteristics of the map representation and differences between the related works. Table 2 presents the main characteristics of path planning and differences between the related works.

**TABLE 1:** Main characteristics of map representation and differences between the related works.

Reference	Metric	Topological	Semantic	Class label/Vocabulary
Shatkay and Kaelbling (2002)	✓	✓	—	—
Rangel et al. (2017)	✓	✓	✓	Preset label
Zheng et al. (2018)	✓	✓	✓	Preset label
Karaoğuz et al. (2016)	✓	—	✓	Preset label
Kostavelis et al. (2016)	✓	✓	✓	Preset label
Luperto and Amigoni (2018)	✓	—	✓	Preset label
Gomez et al. (2020)	✓	✓	✓	Free area or transit area (door)
Rosinol et al. (2021)	✓	✓	✓	Preset label
Hiller et al. (2019)	✓	✓	✓	Preset label
Sousa and Bassani (2022)	✓	—	✓	Preset label
Taniguchi et al. (2017, 2020a)	✓	—	✓	On-site learning (environment-specific words)
SpCoTMHP (Present study)	✓	✓	✓	On-site learning (environment-specific words)

**TABLE 2:** Main characteristics of path planning and differences between the related works.

Reference	Planning approach	Instruction for navigation	Goal determination
Holte et al. (1996)	Classical ( ${\text{A}}^{⋆}$ )	—	Explicitly given as a point
Kostavelis et al. (2016)	Dijkstra and long short-term memory	*go-to* commands through a graphical interface	Explicitly given by the user
Stein et al. (2020)	Learned subgoal planning	—	Explicitly given as a point
Rosinol et al. (2021)	Multilevel ${\text{A}}^{⋆}$	Semantic queries	Explicitly given from queries
Kulkarni et al. (2016), Haarnoja et al. (2018)	Hierarchical reinforcement learning	—	Autonomously estimated
Krantz et al. (2020), Gu et al. (2022), Huang et al. (2023)	Vision and language navigation	Unambiguous and detailed description	Non-explicit (vision based)
Anderson et al. (2018b), Chen et al. (2021)	Deep reinforcement learning	Unambiguous and detailed description	Non-explicit (vision-based)
Taniguchi et al. (2020b)	CaI framework	Daily short speech sentences (containing environment-specific words)	Non-explicit (probabilistic)
SpCoTMHP (Present study)	Hierarchical CaI framework	Daily short speech sentences (containing environment-specific words and waypoints)	Non-explicit (probabilistic)

### 2.1 Topometric semantic mapping

For bridging the topological–geometrical gap, geometrically constrained hidden Markov models have been proposed as probabilistic models for robot navigation in the past (Shatkay and Kaelbling, 2002). The similarity between these models and that proposed in this study is that probabilistic inference is realized for path planning. However, the earlier models do not introduce semantics, such as location names.

Research on semantic mapping has been increasingly emphasized in recent years. In particular, semantic mapping assigns place meanings to the map of a robot (Kostavelis and Gasteratos, 2015; Garg et al., 2020). However, numerous studies have provided preset location labels for areas on a map. For example, LexToMap (Rangel et al., 2017) assigns convolutional neural network (CNN)-recognized lexical labels to a topological map, where the approach enables unsupervised learning based on multimodal perceptual information for categorizing unknown places

The use of topological structures enables more accurate semantic mapping (Zheng et al., 2018); this method is expected to improve performance by introducing topological levels. The nodes in a topological map can vary depending on the methods used, such as room units or small regions (Karaoğuz et al., 2016; Kostavelis et al., 2016; Luperto and Amigoni, 2018; Gomez et al., 2020). Kimera (Rosinol et al., 2021) used multiple levels of spatial hierarchical representation, such as metrics, rooms, places, semantic levels, objects, and agents; here, the robot automatically determined the spatial segmentation unit based on experience.

In several semantic mapping studies (Hiller et al., 2019; Sousa and Bassani, 2022), topological semantic maps were constructed from visual images or metric maps using CNNs. However, these studies have not considered path planning. In contrast, the method proposed herein is characterized by an integrated model that includes learning and planning.

### 2.2 Hierarchical path planning

Hierarchical path planning has been a significant topic of study for long, e.g., hierarchical ${\text{A}}^{⋆}$ (Holte et al., 1996). Using topological maps for path planning (including learning the paths between edges) is more effective for reducing the computational complexity than considering only the movements between cells in a metric map (Kostavelis et al., 2016; Stein et al., 2020; Rosinol et al., 2021). In addition, the extension of map representations to hierarchical semantic maps has enabled navigation based on speech.

Given that the proposed method realizes a hierarchy based on the CaI framework (Levine, 2018), it is theoretically connected with hierarchical reinforcement learning, where the subgoals and policies are estimated autonomously (Kulkarni et al., 2016; Haarnoja et al., 2018). This study investigates tasks similar to hierarchical reinforcement learning to infer the probabilistic models, which are expected to be theoretically readable and integrable with other methods. Vision and language navigation (VLN) aims to help an agent navigate through an environment assisted by natural language instructions while using visual information from the environment (Krantz et al., 2020; Gu et al., 2022; Huang et al., 2023). The present study differs from those on VLNs in several respects. The first difference is in the complexity of the instructions. In VLN tasks, unambiguous and detailed natural language instructions are provided; in contrast, the proposed method involves tasks characterized by the terseness and ambiguity with which people speak daily. The second difference is the training scenario. The VLN dataset uses only common words annotated in advance by people. In contrast, the proposed approach can handle spatial words in communities living in specific environments. The third difference is that although VLNs use vision during path planning, vision was used in the present work to generalize spatial concepts only during training of the proposed method. This is due to the difference between sequential action decisions and global path planning. Finally, deep and reinforcement learning techniques have been used in recent studies on VLNs (Anderson et al., 2018b; Chen et al., 2021); however, the proposed probabilistic model autonomously navigates toward the target location using speech instructions as the modality.

### 2.3 Robotic planning using LLM and foundation models

Recently, there has been growing utilization of LLMs and foundational models for enhancing robot autonomy (Firoozi et al., 2023; Vemprala et al., 2023; Zeng et al., 2023). SayCan (Ahn et al., 2022) integrates pretrained LLMs and behavioral skills to empower the robots to execute context-aware and appropriate actions in real-world settings; in this approach, the LLM conducts higher-level planning based on language while facilitating lower-level action decisions grounded in physical constraints. However, a key challenge remains in accurately capturing the characteristics of the physical space, such as the walls, distances, and room shapes, using only LLMs. In contrast, our study tightly integrates language, spatial semantics, and physical space to estimate the trajectories comprehensively. Furthermore, our proposed method is designed to complement LLM-based planning and natural language processing, with the expectation of seamless integration.

Several studies have employed LLMs and foundational models to accomplish navigation tasks. LM-Nav (Shah et al., 2022) integrates contrastive language–image pretraining (CLIP) (Radford et al., 2021) and generative pretrained transformer-3 (GPT-3) (Brown et al., 2020); this system enables navigation directly through language instructions and robot-perspective images alone. However, this approach necessitates substantial amounts of driving data from the target environment. Conversely, an approach that combines vision–language models (VLMs) and semantic maps has also been proposed. CLIP-Fields (Shafiullah et al., 2023), natural language maps (NLMap) (Chen et al., 2023), and VLMaps (Huang et al., 2023) use LLMs and VLMs to create 2D or 3D spaces and language associations to enable navigation for natural language queries; these approaches mainly record the placements of objects on the map and cannot understand the meanings of the locations or planning for each location. Additionally, LLM/VLM-based approaches have a large common-sense vocabulary similar to an open vocabulary. However, using pretrained place recognizers alone makes it difficult to handle environment-specific names (e.g., Alice’s room). Although LLMs have the potential to handle environment-specific names through in-context learning, they have not been integrated with mapping and navigation in existing models at present. Our spatial concept-based approach addresses knowledge specific to the home environment through on-site learning.

### 2.4 Spatial concept-based approach

In Section 3, we present two major previous studies on which the proposed method is based. As presented in our previous research, SpCoSLAM (Taniguchi et al., 2017, 2020a) forms spatial concept-based semantic maps based on multimodal observations obtained from the environment; here, the multimodal observations for spatial concept formation refer to the images, depth sensor values, odometry, and speech signals. Moreover, the approach can acquire novel place categories and vocabularies from unknown environments. However, SpCoSLAM cannot estimate the topological level, i.e., whether one place is spatially connected with another. The details of the formulation of the probabilistic generative model are described in Supplementary Appendix SA1. The learning procedure for each step is described in Supplementary Appendix SA2. In the present study, we applied the hidden semi-Markov model (HSMM) (Johnson and Willsky, 2013) that estimates the transition probabilities between places and constructs a topological graph instead of the Gaussian mixture model (GMM) used in SpCoSLAM.

In addition, SpCoNavi (Taniguchi et al., 2020b) plans the path in the CaI framework (Levine, 2018) by focusing on the action decisions in the probabilistic generative model of SpCoSLAM. The details on the formulation of CaI are described in Supplementary Appendix SA3. Notably, SpCoNavi realizes navigation from simple speech instructions using a spatial concept acquired autonomously by the robot. However, SpCoNavi does not demonstrate hierarchical path planning, and scenarios specifying a waypoint are not considered. In addition, there are several problems that need to be solved: SpCoNavi based on the Viterbi algorithm (Viterbi, 1967) is computationally expensive given that all the grids of the occupied grid map are used as the state space; it is vulnerable to the real-time performance required for robot navigation; SpCoNavi based on the ${\text{A}}^{⋆}$ approximation has reduced computational cost but inferior performance to that of the Viterbi approach. Therefore, in the present study, we utilized a topological semantic map based on spatial concepts to reduce the number of states and rapidly infer the possible paths among the states.

## 3 Proposed method: SpCoTMHP

We propose the spatial concept-based topometric semantic mapping for hierarchical path planning (SpCoTMHP) approach herein. Spatial concepts refer to categorical knowledge of places from multimodal information obtained through unsupervised learning. The proposed method realizes efficient navigation from human speech instructions through inference based on a probabilistic generative model. The proposed approach also enhances human comprehensibility and explainability for communication by employing Gaussian distributions as the fundamental spatial units (i.e., representing a single place). The capabilities of the proposed generative model are as follows: (i) place categorization by extracting the connection relations between places through unsupervised learning; (ii) many-to-many correspondences between words and places; (iii) efficient hierarchical path planning by introducing two variables ( t and e ) with different time constants.

Three phases can be distinguished in probabilistic generative models: (a) model definition in the probability distribution of the generative process (Section 3.1), (b) inference of the posterior distribution for parameter learning (Section 3.2), and (c) probabilistic inference for task execution after learning (Sections 3.3 and 3.4).

### 3.1 Definition of the probabilistic generative model

**FIGURE 2:**
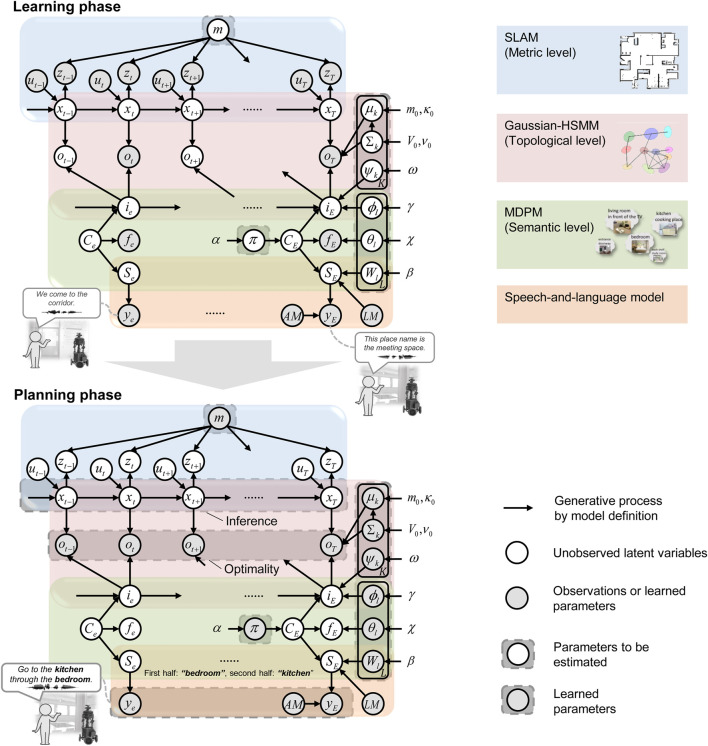
Graphical model representation of the SpCoTMHP (top) spatial concept learning and its path planning phases (bottom). The two phases imply different probabilistic inferences for the same generative model; this has the mathematical advantage that different probabilistic inferences can be applied under the same model assumptions. The integration of several parts into a single model allows the inferences to consider various probabilities throughout. The graphical model represents the conditional dependency between random variables. The gray nodes indicate observations or learned parameters as fixed conditional variables, and white nodes denote unobserved latent variables to be estimated. Arrows from the global variables to local variables other than T and E are omitted. In the learning phase, multimodal observations are obtained several times. Based on these observables, the latent variables are estimated. In the planning phase, the parameters estimated in the learning phase and optimality variables are supplied. Under these conditions, the distribution of trajectories is estimated. $D_{e}$ was omitted from the graphical model representation.

SpCoTMHP is designed as an integrated model for each module: SLAM, HSMM, multimodal Dirichlet process mixture (MDPM) for place categorization, and the speech-and-language model. Therefore, it is simple to distribute the development and further the module coupling in the framework of Neuro-SERKET (Taniguchi et al., 2020c). The integrated model has the advantage of the inference functioning as a whole to complement each uncertainty. Figure 2 presents the graphical model representation of SpCoTMHP, and Table 3 lists each variable of the graphical model. Unlike SpCoSLAM (Taniguchi et al., 2017), SpCoTMHP introduces two different time units (real-time robot-motion-based time step t and event-driven time step e ) and extends the GMM to HSMM. The events represent the timings of user utterances during the learning and switching of locations visited during planning. The generative process (prior distribution or likelihood function) is defined by the graphical model representation of SpCoTMHP.

**TABLE 3:** Descriptions of the random variables used in the proposed model.

Symbol	Definition
m	Environmental map (occupancy grid map)
$x_{t}$	Self-position of the robot (state variable)
$u_{t}$	Control data (action variable)
$z_{t}$	Depth sensor data
$o_{t}$	Optimality variable (event-driven)
$D_{e}$	Duration length for $o_{t}$ in $i_{e}$
$i_{e}\in \left\{k\right\}$	Category index of the position distributions
$C_{e}\in \left\{l\right\}$	Category index of the spatial concepts
$f_{e}$	Visual features of the camera image
$y_{e}$	Speech signal of the uttered sentence
$S_{e}$	Word sequence in the uttered sentence
$\mu_{k}$ , $\Sigma_{k}$	Parameters of multivariate Gaussian distribution (position distribution)
$\psi_{k}$	Parameter of state transitions for $i_{e}$ in $i_{e-1}=k$
π	Parameter of mixture weights for $C_{e}$
$\phi_{l}$	Parameter of mixture weights for $i_{e}$ in $C_{e}=l$
$\theta_{l}$	Parameter of feature distributions for $f_{e}$
$W_{l}$	Parameter of word distributions for $S_{e}$
LM	Language model (n-gram and word dictionary)
AM	Acoustic model for speech recognition
α , β , γ , χ , ω	Hyperparameters of prior distributions
$m_{0}$ , $\kappa_{0}$ , $V_{0}$ , $\nu_{0}$	
T	Final time of robot operation
E	Total number of user utterances (in the learning phase) or total number of location moves (in the planning phase)
L	Total number of spatial concepts
K	Total number of position distributions

SLAM (metric level): The probabilistic generative model of SLAM represents the time-series transition of self-position, and the state space on the map corresponds to the metric level. These probability distributions have been standard in SLAM for probabilistic approaches (Thrun et al., 2005). Accordingly, Eq. (1) represents a measurement model that is a likelihood of a depth sensor $z_{t}$ at a given position $x_{t}$ and map m . Equation (2) represents a motion model that is a state transition related to the position $x_{t}$ based on the action $u_{t}$ in a previous position $x_{t-1}$ in SLAM:

$$z_{t}∼p\left(z_{t}|x_{t},m\right),\;t=1,2,\ldots ,T$$ (1)

$$x_{t}∼p\left(x_{t}|x_{t-1},u_{t}\right).$$ (2)

Here, self-localization assumes a transition at time t due to the motion of the robot. The variable $x_{t}$ is shared with the HSMM.

HSMM (from metric to topological levels): The HSMM can be used to cluster the location data of the robot in terms of position distributions and represent the probabilistic transitions between the position distributions. This refers to transitioning from the metric to topological levels. The HSMM connects two units, namely time t and event e . A binary random variable that indicates whether there is an event is defined as in Eq. 3:

$$\begin{array}{llll} o_{t} & ∼p\left(o_{t}|x_{t},i_{e},\mu ,\Sigma ;\left\{D_{e}\right\}\right)=\left\{\begin{array}{cc} \eta \cdot N\left(x_{t}|\mu_{i_{e}},\Sigma_{i_{e}}\right)\; & \text{if }o_{t}=1,\\ 1-\eta \cdot N\left(x_{t}|\mu_{i_{e}},\Sigma_{i_{e}}\right)\; & \text{if }o_{t}=0, \end{array}\right. & t & =t_{e},\ldots ,t_{e}^{\prime }, \end{array}$$ (3)

where $\eta =1/\sum_{j=1}^{K}N\left(x_{t}|\mu_{j},\Sigma_{j}\right)$ is the normalization constant, N() is a multivariate Gaussian distribution, $\mu =\left\{\mu_{k}\right\}$ , $\Sigma =\left\{\Sigma_{k}\right\}$ , and $o_{t}=1$ is the event that occurred at time t . Here, $o_{t}\in \left\{0,1\right\}$ takes a binary value. This event-driven variable corresponds to the optimality variable in CaI (Levine, 2018). The duration assumes a uniform distribution in [1,T] , as in Eq. 4:

$$\begin{array}{ll} D_{e} & ∼Unif\left(1,T\right),\;e=1,2,\ldots ,E, \end{array}$$ (4)

where the equation relating t and e is $t_{e}=\sum_{e^{\prime }<e}D_{e^{\prime }}$ , and the final time at the event e is $t_{e}^{\prime }=t_{e}+D_{e}-1$ . Thus, E≦T and $T=\sum_{e=1}^{E}D_{e}$ .The position distribution represents a coherent unit of place and is represented by a Gaussian distribution, i.e., as a node in a topological map, where $\mu_{k}$ is a representative point of the node k on the map; $\Sigma_{k}$ represents the spread of the node location k . To capture the transitions between the locations as connection weights between the nodes to represent edges in the topological map, $\psi_{k}$ is introduced as follows, as in Eqs 5–7:

$$\mu_{k}∼N\left(m_{0},\Sigma_{k}/\kappa_{0}\right),\;k=1,2,\ldots ,\infty ,$$ (5)

$$\Sigma_{k}∼IW\left(V_{0},\nu_{0}\right),$$ (6)

$$\psi_{k}∼DP\left(\omega \right),$$ (7)

where IW() is the inverse Wishart distribution, and DP() represents the Dirichlet process (DP). The DP assumes an infinite number of categories and allows infinite mixed HSMMs, thereby enabling learning of the positional distributions, i.e., nodes of a topological map, that flexibly depend on the environment. The inverse Wishart distribution is a conjugate prior distribution on the covariance matrix of the Gaussian distribution. The conjugate prior distribution was established because it allows the posterior distribution to be obtained analytically. Readers are referred to the literature on machine learning (Murphy, 2012) for the specific formulas of these probability distributions.

HSMM + MDPM connection (from topological to semantic levels): The variable $i_{e}$ of the topological node is shared between the HSMM and MDPM. The probability distribution of $i_{e}$ for connecting two modules is defined by unigram rescaling (UR) (Gildea and Hofmann, 1999), as in Eqs 8, 9:

$$i_{e}∼p\left(i_{e}|i_{e-1},\psi ,C_{e},\phi \right)$$ (8)

$$\;\overset{UR}{\approx }\underset{\begin{array}{c} \text{Transition prob.}\\ \text{by HSMM} \end{array}}{\underbrace{Mult\left(i_{e}|\psi_{i_{e-1}}\right)}}\underset{\begin{array}{c} \text{Category dependent term}\\ \text{/ Rescaling term} \end{array}}{\underbrace{\frac{Mult\left(i_{e}|\phi_{C_{e}}\right)}{\sum_{c^{\prime }=1}^{L}Mult\left(i_{e}|\phi_{c^{\prime }}\right)}}},$$ (9)

where $\psi =\left\{\psi_{k}\right\}$ , $\phi =\left\{\phi_{l}\right\}$ , and Mult() is a multinomial distribution. The first term in Eq. (9) denotes the transferability between places, and the second term denotes correspondence between the spatial concept and position distribution. The position distribution $k=i_{e}$ has a high probability when it corresponds to the spatial concept $C_{e}$ and is connected to the position distribution $i_{e-1}$ .

MDPM (semantic level): The MDPM is a mixture distribution model for forming place categories from multimodal observations. Through the spatial concept $l=C_{e}$ , the probabilities of the modalities represented by $\phi_{l}$ , $\theta_{l}$ , and $W_{l}$ are corresponded. The MDPM is positioned at the semantic level, which represents spatial concepts based on places $i_{e}$ , speech–language $S_{e}$ , and image $f_{e}$ features as follows, as in Eqs 10–15:

$$\pi ∼DP\left(\alpha \right),$$ (10)

$$\phi_{l}∼DP\left(\gamma \right),\;l=1,2,\ldots ,\infty ,$$ (11)

$$\theta_{l}∼Dir\left(\chi \right),$$ (12)

$$W_{l}∼Dir\left(\beta \right),$$ (13)

$$C_{e}∼Mult\left(\pi \right),\;e=1,2,\ldots ,E,$$ (14)

$$f_{e}∼Mult\left(\theta_{C_{e}}\right),$$ (15)

where Dir() is the Dirichlet distribution. According to the data, the DP automatically determines the number of spatial concepts L and their position distributions K . A multinomial distribution is applied to the discrete variables; and the Dirichlet distribution and DP are set as the conjugate prior distributions for the multinomial distribution.

MDPM + language model connection (semantic level): The variable of a word sequence $S_{e}$ is shared between the MDPM and language model. The probability distribution of $S_{e}$ for connecting the two modules is defined by UR (Gildea and Hofmann, 1999), as in Eqs 16, 17:

$$S_{e}∼p\left(S_{e}|C_{e},W,LM\right)$$ (16)

$$\;\overset{UR}{\approx }\underset{\text{N}-\text{gram prob.}}{\underbrace{p\left(S_{e}|LM\right)}}\overset{B_{e}}{\underset{b=1}{\prod }}\underset{\begin{array}{c} \text{Category dependent term}\\ \text{/ Rescaling term} \end{array}}{\underbrace{\frac{Mult\left(S_{e,b}|W_{C_{e}}\right)}{\sum_{c^{\prime }=1}^{L}Mult\left(S_{e,b}|W_{c^{\prime }}\right)}}},$$ (17)

where $W=\left\{W_{l}\right\}$ . Moreover, $B_{e}$ is the number of words in the sentence, and $S_{e,b}$ is the b -th word in the sentence at event e . The first term in Eq. (17) is the probability of occurrence of a word based on the n-gram language model LM . Specifically, $p\left(S_{e}|LM\right)=\prod_{b=1}^{B_{e}}p\left(S_{e,b}|S_{e,b-n+1:b-1};LM\right)$ . The second term is the spatial concept-dependent word probability distribution, which is computed independently for each word.

Speech-and-language model: The generative process for the likelihood of speech given a word sequence is, as in Eq. 18:

$$\begin{array}{ll} y_{e} & ∼p\left(y_{e}|S_{e},AM\right). \end{array}$$ (18)

This probability distribution does not usually appear explicitly but is internalized as an acoustic model in probability-based speech recognition systems.

### 3.2 Spatial concept learning as topometric semantic mapping

The joint posterior distribution is described as

$$p\left(x_{0:T},S_{1:E},C_{1:E},\Theta |u_{1:T},z_{1:T},o_{t_{1:E}^{\prime }}^{*},y_{1:E},f_{1:E},h\right),$$ (19)

where $C_{1:E}=\left\{i_{1:E},C_{1:E}\right\}$ denotes the set of latent variables, Θ={m,μ,Σ,ψ,π,ϕ,θ,W,LM,AM} denotes the set of global model parameters, and $h=\left\{\alpha ,\beta ,\gamma ,\chi ,\omega ,m_{0},\kappa_{0},V_{0},\nu_{0}\right\}$ denotes the set of hyperparameters. The set of event-driven variables is given by $o_{t_{1:E}^{\prime }}^{*}={\left\{o_{t_{e}^{\prime }}=1\right\}}_{e=1}^{E}$ .

In this paper, as an approximation to sampling from Eq. (19), the parameters are estimated as follows:

$$x_{0:T},m∼p\left(x_{0:T},m|u_{1:T},z_{1:T}\right),$$ (20)

$$S_{e}∼p\left(S_{e}|y_{e},LM,AM\right),\;e=1,2,\ldots ,E,$$ (21)

$$C_{1:E},\Theta^{\prime }∼p\left(C_{1:E},\Theta^{\prime }|x_{0:T},o_{t_{1:E}^{\prime }}^{*},S_{1:E},f_{1:E},h\right),$$ (22)

where $\Theta^{\prime }=\left\{\mu ,\Sigma ,\psi ,\pi ,\phi ,\theta ,W\right\}$ . Equation (20) is realized using grid-based FastSLAM 2.0 (Grisetti et al., 2007), and Eq. (21) represents the speech recognition of $y_{e}$ . Here, LM and AM were preset. The proposed method then handles uncertainties in speech recognition by capturing the N -best speech recognition results as Monte Carlo approximations. The variables in Eq. (22) can be learned using Gibbs sampling, which is a Markov-chain Monte-Carlo-based batch learning algorithm, specifically the weak-limit and direct-assignment sampler (Johnson and Willsky, 2013).

In the learning phase, the user provides a teaching utterance each time the robot transitions between locations. Given that the utterance is event-driven, it is assumed that the variables for the spatial concepts are observed only at event e . Here, the time of the e -th event (when the robot observes that an utterance indicates a place) is $t_{e}^{\prime }$ . In particular, $o_{t_{e}^{\prime }}=1$ is observed at the instants of $t_{e}^{\prime }$ , and $o_{t}$ is unobserved at other times. Therefore, the inference for learning $i_{e}$ is equivalent to a HMM.

Reverse replay: In the case of spatial movements, we can transition from $i_{e-1}$ to $i_{e}$ or *vice versa*. Therefore, $i_{E:1}^{\prime }$ , which is replayed using the steps of e in reverse order, can be used for learning when sampling ψ . This is based on the replay performed in the hippocampus of the brain (Foster and Wilson, 2006).

### 3.3 Hierarchical path planning by control as inference

The probabilistic distribution, which represents the trajectory $\tau =\left\{u_{1:T},x_{1:T}\right\}$ when a speech instruction $y_{e}$ is given, is maximized to estimate an action sequence $u_{1:T}$ (and the path $x_{1:T}$ on the map) as follows:

$$u_{1:T}=\underset{u_{1:T}}{\mathrm{arg max}}p\left(\tau |o_{1:T}^{*},y_{1:E},x_{0},\Theta \right).$$ (23)

The planning horizon at the metric level T is the final time of the entire task when a one-time step traverses one grid block on the metric map. The planning horizon at the topological level E is the number of event steps used to navigate by speech instruction. As shown in Eqs. (3, 4), each event step e corresponds to the time series $t_{e}:t_{e}^{\prime }$ . The metric-level planning horizon in Step e corresponds to the duration $D_{e}$ of the HSMM. In the metric-level planning horizon, the event-driven variable is always $o_{1:T}^{*}={\left\{o_{t}=1\right\}}_{t=1}^{T}$ by the CaI. The speech instruction $y_{e}$ is assumed to be the same as that from e=1 to E . This indicates that $o_{t}$ and $y_{e}$ are multiple optimals in terms of the CaI (Kinose and Taniguchi, 2020). From the above, Eq. (23) is rewritten as follows:

$$\begin{array}{l} p\left(\tau |o_{1:T}^{*},y_{1:E},x_{0},\Theta \right)\approx \overset{E}{\underset{e=1}{\prod }}\left[\overset{K}{\underset{i_{e}=1}{\sum }}\frac{Mult\left(i_{e}|\psi_{i_{e-1}}\right)}{\sum_{c^{\prime }=1}^{L}Mult\left(i_{e}|\phi_{c^{\prime }}\right)}\right.\\ \;\overset{L}{\underset{C_{e}=1}{\sum }}Mult\left(i_{e}|\phi_{C_{e}}\right)Mult\left(S_{e}|W_{C_{e}}\right)Mult\left(C_{e}|\pi \right)\\ \;\left.\overset{t_{e}^{\prime }}{\underset{t=t_{e}}{\prod }}N\left(x_{t}|\mu_{i_{e}},\Sigma_{i_{e}}\right)p\left(x_{t}|m\right)p\left(x_{t}|x_{t-1},u_{e}\right)\right], \end{array}$$ (24)

$$S_{e}∼p\left(S_{e}|y_{e},LM,AM\right),$$ (25)

where $p\left(x_{t}|m\right)$ is a probabilistic representation of the cost map, and $D_{1:E}$ is the maximum limit value given. In addition, the word sequence $S_{e}$ is obtained by speech recognition of $y_{e}$ as the N -best bag of words, in Eq. 25. The assumptions, such as the SLAM models and cost map, in the derivation of the equation are the same as those used for SpCoNavi (Taniguchi et al., 2020b).

In the present study, we assumed that the robot could extract words indicating the goal and waypoint from a particular sentence utterance. In topological-level planning including the waypoint, the waypoint word is input in the first half while the target word is presented in the second half of the utterance.

### 3.4 Approximate inference for hierarchical path planning

The strict inference of Eq. (24) requires a double-forward backward calculation. In this case, reducing the calculation cost is necessary to accelerate path planning, which is one of the objectives of this study. Therefore, we propose an algorithm to solve Eq. (24). Algorithm 1 presents the hierarchical planning approach as produced by SpCoTMHP. Here, the path planning is divided into topological and metric levels, and the CaI is solved at each level. Metric-level planning assumes that the partial paths in each of the transitions between places are solved in ${\text{A}}^{⋆}$ . The partial paths can be precomputed regardless of the speech instructions. Topological-level planning is approximated using the probability distribution of $i_{e}$ by assuming Markov transitions. Finally, the partial paths in each of the transitions between places are integrated as a complete path. Thus, metric and topological planning can influence each other.

Algorithm 1 Hierarchical path planning algorithm.

1:  //Precalculation:
2: $\left\{{\hat{x}}_{t_{e}^{\prime }|i_{e}}\right\}∼Gaussian\_Mixture\left(\phi ,\mu ,\Sigma \right)$

3:  Create a graph between the waypoint candidates
4:  for all nodes, $n_{i_{e-1}}\to n_{i_{e}}$ , do
5: ${\hat{x}}_{i_{e-1},i_{e}}^{\left[n_{i_{e-1}},n_{i_{e}}\right]}\leftarrow A^{⋆}\left({\hat{x}}_{t_{e-1}^{\prime }|i_{e-1}}^{\left[n_{i_{e-1}}\right]},{\hat{x}}_{t_{e}^{\prime }|i_{e}}^{\left[n_{i_{e}}\right]},w_{e}\right)$

6:   Calculate likelihoods ${\hat{w}}_{i_{e-1},i_{e}}^{\left[n_{i_{e-1}},n_{i_{e}}\right]}$ for the partial paths
7:  end for
8:  //When a speech instruction $y_{e}$ is given:
9: $S_{e}\leftarrow Speech\_Recognition\left(y_{e},LM,AM\right)$

10:  Estimate an index $i_{0}$ of the place in the initial position $x_{0}$

11: $n_{1:E},i_{1:E}\leftarrow Search\left(i_{0},S_{e},\hat{w},\Theta \right)$ //Eq. (28)
12:  Connect the partial paths $n_{1:E}$ as the complete path $x_{1:E}$

13: $x_{1:E}\leftarrow Path\_Smoothing\left(x_{1:E},m\right)$ //optional process

Path planning at the metric level (i.e., partial path $x_{i_{e-1},i_{e}}$ when transitioning from $i_{e-1}$ to $i_{e}$ ) is described as follows:

$$\begin{array}{ll} x_{t_{e}:t_{e}^{\prime }} & =\underset{x_{t_{e}:t_{e}^{\prime }}}{\mathrm{arg max}}\overset{t_{e}^{\prime }}{\underset{t=t_{e}}{\prod }}N\left(x_{t}|\mu_{i_{e}},\Sigma_{i_{e}}\right)\\ & \;\;\;p\left(x_{t}|m\right)p\left(x_{t}|x_{t-1},u_{t}\right). \end{array}$$ (26)

This indicates that a metric-level path inference can be expressed in terms of the CaI.

Calculating Eq. (24) for all possible positions was difficult. Therefore, we used the mean or sampled values from the Gaussian mixture of position distributions as the goal position candidates, i.e., ${\hat{x}}_{t_{e}^{\prime }|i_{e}}^{\left[n_{i_{e}}\right]}∼N\left(x_{t}|\mu_{i_{e}},\Sigma_{i_{e}}\right)$ . Here, $n_{i_{e}}$ is an index that takes values of up to $N_{i_{e}}$ , which is the number of candidate points sampled for a specific $i_{e}$ . By sampling multiple points according to the Gaussian distribution, the candidate waypoints that follow the rough shape of the place can be selected. For example, the robot does not necessarily have to go to the center of a lengthy corridor.

Therefore, as a concrete solution to Eq. (26), the partial paths in the transitions of the candidate points from place $i_{e-1}$ to place $i_{e}$ are estimated as follows, as in Eq. 27:

$${\hat{x}}_{i_{e-1},i_{e}}^{\left[n_{i_{e-1}},n_{i_{e}}\right]}=A^{⋆}\left({\hat{x}}_{t_{e-1}^{\prime }|i_{e-1}}^{\left[n_{i_{e-1}}\right]},{\hat{x}}_{t_{e}^{\prime }|i_{e}}^{\left[n_{i_{e}}\right]},w_{e}\right),$$ (27)

where $A^{⋆}\left(s,g,w_{e}\right)$ denotes the function of the ${\text{A}}^{⋆}$ search algorithm, s is the initial position, g is the goal position, and $w_{e}=N\left(x_{t}|\mu_{i_{e}},\Sigma_{i_{e}}\right)p\left(x_{t}|m\right)$ is the cost function. The estimated partial path length can then be interpreted as the estimated value of $D_{e}$ .

The selection of a series of partial metric path candidates corresponds to the selection of the entire path. Thus, we can replace the formulation of the maximization problem of Eq. (24) with that of Eq. (28). Each partial metric path has corresponding indices $i_{e-1}$ and $i_{e}$ . Therefore, given a series of index pairs representing transitions between the position distributions, the candidate paths to be considered can naturally be narrowed down to a series of corresponding partial paths. The series of candidate indices that determines the series of candidate paths is thus $n_{1:E}=\left(n_{i_{0}},n_{i_{1}},\ldots ,n_{i_{E}}\right)$ in this case. This partial path sequence can be regarded as a sampling approximation of $x_{1:T}$ .

By taking the maximum value instead of the summation $i_{1:E}$ , path planning at the topological level can be described as

$$\begin{array}{ll} n_{1:E},i_{1:E} & =\underset{n_{1:E},i_{1:E}}{\mathrm{arg max}}\overset{E}{\underset{e=1}{\prod }}\frac{Mult\left(i_{e}|\psi_{i_{e-1}}\right)}{\sum_{c^{\prime }=1}^{L}Mult\left(i_{e}|\phi_{c^{\prime }}\right)}{\hat{w}}_{i_{e-1},i_{e}}^{\left[n_{i_{e-1}},n_{i_{e}}\right]}\\ & \;\overset{L}{\underset{C_{e}=1}{\sum }}Mult\left(i_{e}|\phi_{C_{e}}\right)Mult\left(S_{e}|W_{C_{e}}\right)Mult\left(C_{e}|\pi \right), \end{array}$$ (28)

where ${\hat{w}}_{i_{e-1},i_{e}}^{\left[n_{i_{e-1}},n_{i_{e}}\right]}$ is the likelihood of the metric path ${\hat{x}}_{i_{e-1},i_{e}}^{\left[n_{i_{e-1}},n_{i_{e}}\right]}$ when transitioning from a candidate place point $i_{e-1}$ to the next candidate place point $i_{e}$ at Step e . In this case, it is equivalent to formulating the state variables in the distribution for the CaI as $x_{1:E}$ and $i_{1:E}$ . Therefore, path planning at the topological level can be expressed as the CaI at the event step e .

## 4 Experiment I: planning tasks in a simulator

We experimented with path planning using spatial concepts by including topological structures via human speech instructions. In this experiment, as a first step, we demonstrated that the proposed method improves the efficiency of path planning when the ideal spatial concept is used. The simulator environment was SIGVerse Version 3.0 (Inamura and Mizuchi, 2021), and the virtual robot model used was the Toyota HSR. We used five three-bedroom home environments^3^ with different layouts and room sizes.

### 4.1 Spatial concept-based topometric semantic map

**FIGURE 3:**
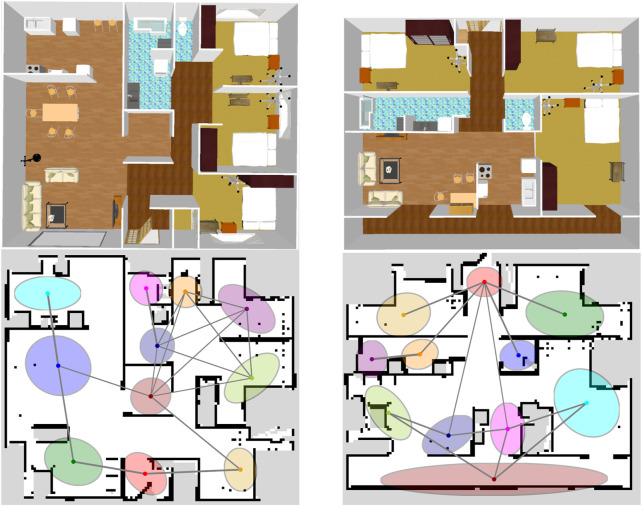
Overhead view of the simulator environments (top) and ideal spatial concepts expressed by SpCoTMHP on the environmental map (bottom) in Experiment I. The colors of the position distributions were randomly set. If $\left(\psi_{k_{1},k_{2}}+\psi_{k_{1},k_{2}}\right)/2>1/K$ , the centers $\mu_{k_{1}},\mu_{k_{2}}$ of the Gaussian distributions are connected by an edge. This means that the edges are drawn only if the average transition probabilities from $k_{1}$ to $k_{2}$ and $k_{2}$ to $k_{1}$ are higher than the uniform transition probability.

There were 11 spatial concepts and position distributions for each environment (Figure 3 bottom; Supplementary Appendix SA4). Fifteen utterances were provided by the user for each place as the training data. The SLAM and speech recognition modules were inferred individually by splitting from the model, i.e., the self-location $x_{1:E}$ and word sequence $S_{1:E}$ were input to the model as observations. An environment map was generated by the *gmapping* package that implements grid-based FastSLAM 2.0 (Grisetti et al., 2007) in the robot operating system (ROS). In this experiment, a word dictionary was prepared in advance for the vocabulary to be used by considering the focus as evaluation of path planning. In addition, we assumed that the speech recognition results were obtained accurately. The model parameters for the spatial concept were obtained via sampling from a conditional distribution, i.e., Eq. (22). We adopted the ideal learning results of the spatial concepts, and the latent variables $C_{t}$ and $i_{t}$ were obtained accurately. Figure 3 presents two examples of the overhead views of the home environments built into the simulator and their spatial concepts (i.e., position distributions and their connections) in the environmental maps.

### 4.2 Path planning from speech instructions

Two types of path planning tasks were performed in the experiments, which included a variation where the waypoints and goals were recombined at different places. The waypoint and goal words in user instructions were extracted by a simple natural language process and entered into the model as $\left\{S_{e}\right\}$ . Basic task: The robot obtained the words identifying the target locations as instructions, e.g., “*Go to the bedroom.*”Advanced task: The robot obtained the words identifying the waypoint locations and targets as speech instructions, such as “*Go to the bedroom via the corridor.*” We supplied both the waypoint and target words as bag of words to SpCoNavi as this task was not demonstrated previously (Taniguchi et al., 2020b).

We compared the performances of the methods as follows:

(A) ${\text{A}}^{⋆}$ algorithm (goal estimated by spatial concepts): the goal position was obtained as $x^{*}∼p\left(x|S^{*},\Theta \right)$ in SpCoSLAM using the speech recognition results $S^{*}$ .
(B) SpCoSLAM (Taniguchi et al., 2017) + SpCoNavi (Taniguchi et al., 2020b) with the Viterbi algorithm (Viterbi, 1967).
(C) SpCoSLAM (Taniguchi et al., 2017) + SpCoNavi (Taniguchi et al., 2020b) with ${\text{A}}^{⋆}$ approximation.
(D) Hierarchical path planning without CaI, similar to Niijima et al. (2020): the goal nodes were estimated by $i_{e}∼p\left(i_{e}|S^{*},\Theta \right)$ . The topological planning used heuristic costs as the (I) cumulative cost and (II) distances of partial paths in ${\text{A}}^{⋆}$ .
(E) SpCoTMHP (topological level: Dijkstra, metric level: ${\text{A}}^{⋆}$ )

The evaluation metrics for path planning include the success weighted by path length (SPL) (Anderson et al., 2018a) when the robot reaches the target location and calculated runtime in seconds (time). The N-SPL is the weighted success rate when the robot reaches the closest target from the initial position for several places having the same name. The W-SPL is the weighted success rate when the robot passes the correct waypoints. The WN-SPL is the weighted success rate when the robot reaches the closest target by passing the correct waypoints; the WN-SPL is the overall measure of path planning efficiency in advanced tasks.

Conditions: The planning horizons were E=10 for the topological level and D=100 as the maximum limit for the metric level in SpCoTMHP. The number of position candidates in the sample was $N_{i_{e}}=1$ ^4^. The proposed method subjected the paths to moving average smoothing with a window size of 5. The planning horizon of SpCoNavi was T=200 . The number of goal candidates for SpCoNavi ( ${\text{A}}^{⋆}$ approximation) was J=10 . The parameters E , D , and T were large enough for the complexity of the environment, and J was the same as in the original experimental setting (Taniguchi et al., 2020b). The global cost map was obtained from the *costmap_2d* package in the ROS. The robot’s initial position was set from arbitrary movable coordinates on the map, and the user provided a word to indicate the target name. The state of self-position $x_{t}$ was expressed discretely for each movable cell in the occupancy grid map m . The motion model was a simple deterministic model, i.e., $x_{t}=x_{t-1}+u_{t}$ . In other words, motion errors were not assumed in the path planning. The control value $u_{t}$ was assumed to move by a single cell on the map for each time step, and the action $u_{t}$ was discretized as A= {stay, up, down, left, right}. The simulations were implemented in Python on one central processing unit (CPU) with an Intel Core i7-6850K having 16 GB DDR4 2133-MHz synchronous dynamic random-access memory (SDRAM).

**FIGURE 4:**
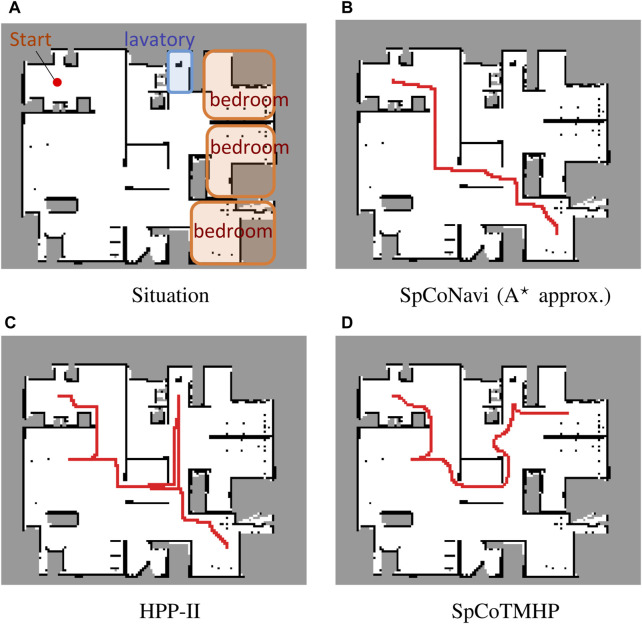
Example of path planning in the advanced task. The instruction: “*Go to the* ***bedroom*** *via the* ***lavatory***” (Experiment I).

Results: Tables 4 and 5 present the evaluation results for the basic and advanced planning tasks. Figure 4 presents an example of the estimated path^5^. Overall, SpCoTMHP outperformed the comparison methods and had significantly reduced computation times. The basic task demonstrated that the proposed method could solve the problem of stopping along the path before reaching the objective, which occurs in SpCoSNavi ( ${\text{A}}^{⋆}$ approximation). The N-SPL of the baseline methods were lower than that of the proposed method because there were cases where the goal was selected as a bedroom far from the initial position (Figures 4B, C). This demonstrated the effectiveness of the proposed method based on probabilistic inference (i.e., CaI).

**TABLE 4:** Evaluation results for path planning in the basic task (Experiment I).

Method	Hierarchy	CaI	**SPL** ↑	**N-SPL** ↑	**Time** ↓
${\text{A}}^{⋆}$	-	-	0.570	0.463	$9.47\times 10^{0}$
SpCoNavi (Viterbi)	-	✓	**0.976**	**0.965**	$2.68\times 10^{3}$
SpCoNavi ( ${\text{A}}^{⋆}$ approximation)	-	✓	0.404	0.388	$5.42\times 10^{1}$
HPP-I (path cost)	✓	-	0.723	0.605	7.56 × 10^0^
HPP-II (path distance)	✓	-	0.714	0.571	$7.96\times 10^{0}$
SpCoTMHP	✓	✓	0.861	0.812	4.79 × 10^0^

Bold indicates the best evaluation value among the methods compared.

**TABLE 5:** Evaluation results for path planning in the advanced task (Experiment I).

Method	Hierarchy	CaI	**SPL** ↑	**W-SPL** ↑	**N-SPL** ↑	**WN-SPL** ↑	**Time** ↓
${\text{A}}^{⋆}$	-	-	0.312	0.449	0.233	0.034	$9.44\times 10^{0}$
SpCoNavi ( ${\text{A}}^{⋆}$ approximation)	-	✓	0.266	0.308	0.252	0.013	$5.53\times 10^{1}$
HPP-I (path cost)	✓	-	0.917	0.248	0.773	0.191	7.53 × 10^0^
HPP-II (path distance)	✓	-	0.902	0.250	0.729	0.183	$8.03\times 10^{0}$
SpCoTMHP	✓	✓	**0.922**	**0.906**	**0.794**	**0.781**	0.39 × 10^0^

Bold indicates the best evaluation value among the methods compared.

The advanced task confirmed that the proposed method could estimate the path via the waypoint (Figure 4D). Although SpCoTMHP had the disadvantage of estimating slightly redundant paths, the reduced computation time and improved planning performance render it a more practical approach than the conventional methods. Consequently, the proposed method achieved better path planning by considering the initial, waypoint, and goal positions.

SpCoTMHP exhibited faster path planning than SpCoNavi (Viterbi) despite its inferior performance in the basic path planning task. This improvement stems from the reduced number of inference states and computational complexity achieved through hierarchization and approximation. In both the basic and advanced tasks, SpCoTMHP notably enhanced the path planning performance over SpCoNavi ( ${\text{A}}^{⋆}$ approximation). Consequently, the SpCoNavi problem outlined in Section 2.4 was effectively addressed by SpCoTHMP.

## 5 Experiment II: real environment

We demonstrated that the formation of spatial concepts, including topological relations between places, could also be realized in a real-world environment. Real-world datasets are more complex and involve more uncertainties than simulators. Therefore, as detailed in Section 5.1, we first confirmed that the proposed method had improved learning performance over the conventional method SpCoSLAM. Thereafter, as detailed in Section 5.2, we determined the impacts of the spatial concept parameters learned in Section 5.1 on the inference of path planning. Additionally, we confirmed that the proposed method could plan a path based on the learned topometric semantic map.

### 5.1 Spatial concept-based topometric semantic mapping

Conditions: The experimental environment was identical to that in the open dataset albert-b-laser-vision^6^, which was obtained from the robotics dataset repository (Radish) (Stachniss, 2003). The details of the dataset are shown in Supplementary Appendix SA5. The utterances included 70 sentences in Japanese, such as “*The name of this place is student workroom*,” “*You can find the robot storage space here*,” and “*This is a white shelf*.” The hyperparameters for learning were set as follows: α=0.5 , γ=0.05 , β=0.1 , χ=1.0 , ω=0.5 , $m_{0}={\left[0,0\right]}^{\text{T}}$ , $\kappa_{0}=0.001$ , $V_{0}=diag\left(2,2\right)$ , and $\nu_{0}=3$ . The parameters were set empirically within the typical ranges with reference to SpCoSLAM (Taniguchi et al., 2017, 2020a). The other settings were identical to those in Experiment I.

Evaluation metrics: Normalized mutual information (NMI) (Kvalseth, 1987) and adjusted Rand index (ARI) (Hubert and Arabie, 1985), which are the most widely used metrics in clustering tasks for unsupervised learning, were used as the evaluation metrics for learning the spatial concepts. The NMI was obtained by normalizing the mutual information between the clustering results and correct labels in the range of 0.0–1.0. Moreover, the ARI is 1.0 when the clustering result matches the correct label and 0.0 when it is random. The time taken for learning was additionally recorded as a reference value.

**FIGURE 5:**
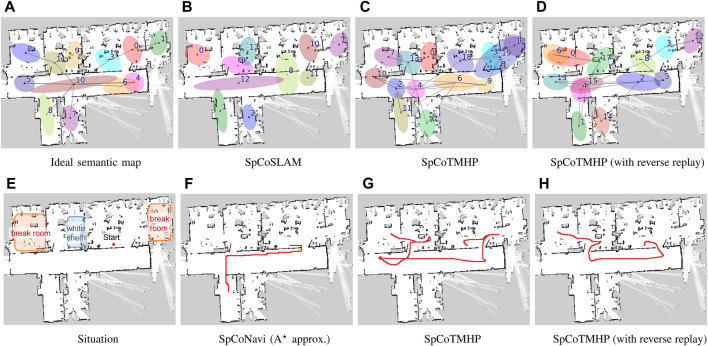
Top **(A–D)**: Results of spatial concept learning. Bottom **(E–H)**: Results of path planning. The speech instruction provided was “*Go to the* ***break room*** *via the* ***white shelf***.” The *break room* was taught in two rooms: upper right and upper left corners. The *white shelf* is in the second room from the left on the upper half of the map (Experiment II).

Results: Figures 5A–D present an example of spatial concept learning. For example, the map in Figure 5C caused overlapping distributions in the upper right corner and skipped connections to neighboring distributions, which were mitigated by the map in Figure 5D. Table 6 presents the evaluation results from the average of ten trials of spatial concept learning. SpCoTMHP achieved a higher learning performance (i.e., NMI and ARI values) than SpCoSLAM, indicating that the categorization of spatial concepts and position distributions was more accurate when considering the connectivity of the places. In addition, the proposed method with reverse replay demonstrated the highest performance. Consequently, using both place transitions during learning and *vice versa* may be useful for learning spatial concepts. Moreover, Table 6 shows that there was no significant difference in the computation time of the learning algorithm.

**TABLE 6:** Learning performances for spatial concepts and position distributions, as well as computation times of the learning algorithms (Experiment II).

	**NMI** ↑		**ARI** ↑		**Time** ↓
**Methods**	$C_{e}$	$i_{e}$	$C_{e}$	$i_{e}$	(sec.)
SpCoSLAM	0.767	0.803	0.539	0.578	$1.28\times 10^{2}$
SpCoTMHP	0.779	0.858	0.540	0.656	$1.33\times 10^{2}$
SpCoTMHP (with reverse replay)	**0.786**	**0.862**	**0.562**	**0.658**	$1.29\times 10^{2}$

Bold indicates the best evaluation value among the methods compared.

### 5.2 Path planning from speech instructions

The speech instruction provided was “*Go to the break room via the white shelf,*” and all other settings were identical to those in Experiment I. Figures 5E–H present the results for path planning using the spatial concepts. Although SpCoSLAM could not reach the waypoint and goal in the map of Figure 5F, SpCoTMHP could estimate the path to reach the goal via the waypoint in the maps in Figures 5G, H. The learning with reverse replay in the map of Figure 5D shortened the additional route that would have resulted from the transition bias between places during learning in the map of Figure 5C. The failure observed in Figure 5F with SpCoNavi using waypoints is primarily attributed to the inputs with names of the given locations, regardless of these being waypoints or goals, in the bag-of-words format. The results revealed that the proposed method performs hierarchical path planning accurately, although the learning results are incomplete, as shown in Table 6. As a reference, the inference times for path planning were $1.02\times 10^{3}$ s for SpCoNavi, $3.97\times 10^{-2}$ s for SpCoTMHP, and $2.39\times 10^{-2}$ s for SpCoTMHP (with reverse replay). The results of Experiment I (Section 4) thus demonstrate the computational efficiency of the proposed hierarchical path planning.

## 6 Conclusion

We achieved topometric semantic mapping based on multimodal observations and hierarchical path planning through waypoint-guided instructions. The experimental results demonstrated improved performance for spatial concept learning and path planning in both simulated and real-world environments. Additionally, the approximate inference achieved high computational efficiency regardless of the model complexity.

Although these are encouraging results, our study has a few limitations as follows:

1. Scalability: The experiments assumed a single waypoint; however, the proposed method can theoretically handle multiple waypoints. Although the computational complexity increases with the topological planning horizon E , scalability will be sufficiently ensured when the users only require a few waypoints. In practical scenarios, one or two waypoints are highly probable in daily life.
2. Instruction variability: A typical instruction representation was used in the experiment. As a preprocessing step, LLMs can be used to handle instruction variability (Shah et al., 2022).
3. Redundant waypoints: Our approach may require passing through redundant waypoints, even if visiting the waypoint itself is unnecessary. For instance, in Figure 5, if it were possible to directly specify “the break room next to the white shelf,” there would be no need to pass by the white shelf as a waypoint. In such cases, extending the system to an open-vocabulary LLM-based semantic map could provide a viable solution.
4. Path restrictions: The paths generated by the proposed model are restricted by the transition probabilities between the locations encountered during training. In contrast, the model by Banino et al. (2018) can navigate through paths that are not traversed during training. Exploring the integration of such vector-based navigation techniques with our spatial concept-based approach could potentially enable shorter navigation while enhancing the model’s flexibility and robustness.

Future research on the proposed approach will therefore include utilizing common-sense reasoning (Hasegawa et al., 2023), such as foundation models and transfer of knowledge (Katsumata et al., 2020) with respect to the spatial adjacencies across multiple environments. In this study, we trained the model using the procedure described in Section 3.2. Simultaneous and online learning for the entire model can also be realized with particle filters (Taniguchi et al., 2017). The proposed method was found to be computationally efficient, thus rendering it potentially applicable to online path planning, such as model predictive control (Stahl and Hauth, 2011; Li et al., 2019). Additionally, the proposed model has the potential for visual navigation and generation of linguistic path explanations through cross-modal inference by the robot.

## Data Availability

The raw data supporting the conclusions of this article will be made available by the authors, without undue reservation.

## Footnotes

1 Alternatively, learning can be realized by active exploration based on generating questions or image captioning (Mokady et al., 2021) for the user (Ishikawa et al., 2023; Taniguchi et al., 2023). For example, the robot asks questions such as “*What kind of place is this?*” to the users.

2 The source code is available at https://github.com/a-taniguchi/SpCoTMHP.git.

3 Three-dimensional (3D) home environment models are available at https://github.com/a-taniguchi/SweetHome3D_rooms.

4 This means a one-sample approximation to the candidate waypoints for the partial path. A related description can be found in Section 3.4. A one-sample approximation will be sufficient if the Gaussian distributions representing the locations and their transitions are obtained accurately.

5 A video of the robot simulation moving along the estimated path is available at https://youtu.be/w8vfEPtnWEg.

6 The dataset is available at https://dspace.mit.edu/handle/1721.1/62291.
