# Supplementary material for: Hierarchical path planning from speech instructions with spatial concept-based topometric semantic mapping
Source: Front Robot AI. 2024 Aug 1;11:1291426. doi: 10.3389/frobt.2024.1291426 (PMC11324419; doi:10.3389/frobt.2024.1291426)
Supplement: Supplementary file 1 [file Supplementaryfile1.pdf]

# Supplementary Material

## 1 FORMULATION OF THE GENERATIVE PROCESS OF SPCOSLAM AND SPCONAVI

The details of the formulation of the generative process represented by the graphical model of SpCoSLAM and SpCoNavi can be described as follows:

$$\pi \sim \mathrm{DP}(\alpha) \tag{S1}$$
$$\phi_l \sim \mathrm{DP}(\gamma) \tag{S2}$$
$$\theta_l \sim \mathrm{Dir}(\chi) \tag{S3}$$
$$W_l \sim \mathrm{Dir}(\beta) \tag{S4}$$
$$LM \sim p(LM \mid \lambda) \tag{S5}$$
$$\Sigma_k \sim \mathcal{IW}(V_0, \nu_0) \tag{S6}$$
$$\mu_k \sim \mathcal{N}(m_0, \Sigma_k/\kappa_0) \tag{S7}$$
$$x_t \sim p(x_t \mid x_{t-1}, u_t) \tag{S8}$$
$$z_t \sim p(z_t \mid x_t, m) \tag{S9}$$
$$C_t \sim \mathrm{Mult}(\pi) \tag{S10}$$
$$i_t \sim p(i_t \mid x_t, \boldsymbol{\mu}, \boldsymbol{\Sigma}, \boldsymbol{\phi}, C_t) \tag{S11}$$
$$f_t \sim \mathrm{Mult}(\theta_{C_t}) \tag{S12}$$
$$S_t \sim p(S_t \mid \mathbf{W}, C_t, LM) \tag{S13}$$
$$y_t \sim p(y_t \mid S_t, AM) \tag{S14}$$

where $\mathrm{DP}()$ represents the Dirichlet process, $\mathrm{Dir}()$ is the Dirichlet distribution, $\mathcal{IW}()$ is the inverse–Wishart distribution, $\mathcal{N}()$ is the multivariate normal distribution, and $\mathrm{Mult}()$ is the multinomial distribution. See (Murphy, 2012) for the specific formulas of the above probability distributions.

The probability distribution of Equation (S8) represents a motion model, i.e., a state transition model, in SLAM. The probability distribution of Equation (S9) represents a measurement model in SLAM.

The probability distribution of Equation (S11) can be defined as

$$p(i_t \mid x_t, \boldsymbol{\mu}, \boldsymbol{\Sigma}, \boldsymbol{\phi}, C_t) = \frac{\mathcal{N}(x_t \mid \mu_{i_t}, \Sigma_{i_t})\mathrm{Mult}(i_t \mid \phi_{C_t})}{\sum_{i_t=j} \mathcal{N}(x_t \mid \mu_j, \Sigma_j)\mathrm{Mult}(j \mid \phi_{C_t})}. \tag{S15}$$

The probability distribution of Equation (S13) is approximated by unigram rescaling (Gildea and Hofmann, 1999), as

$$p(S_t \mid \mathbf{W}, C_t, LM) \approx p(S_t \mid LM) \prod_{B_t} \frac{\mathrm{Mult}(S_{t,b} \mid W_{C_t})}{\sum_{c'} \mathrm{Mult}(S_{t,b} \mid W_{c'})}, \tag{S16}$$

where $B_t$ denotes the number of words in the sentence and $S_{t,b}$ is $b$-th word in the sentence at the time-step of $t$.

## 2 FORMULATION AND PROCEDURE FOR EACH STEP OF THE ONLINE LEARNING ALGORITHM

The online learning algorithm introduces sequential equation updates to estimate the parameters of the spatial concepts into the formulation of a Rao–Blackwellized particle filter (Doucet et al., 2000) in the FastSLAM 2.0 (Montemerlo et al., 2003) and its grid-based SLAM (Grisetti et al., 2007). The particle filter is advantageous, in that parallel processing can be readily applied because the calculations concerning the particles can be calculated independently. Theoretically, other particle-filter-based SLAMs, besides FastSLAM 2.0, can also be used.

In the formulation of SpCoSLAM, the joint posterior distribution can be factorized to the probability distributions of a language model $LM$, a map $m$, the set of model parameters of spatial concepts $\Theta = \{\mathbf{W}, \boldsymbol{\mu}, \boldsymbol{\Sigma}, \theta, \phi, \pi\}$, the joint distribution of the self-positions $x_{0:t}$, and the set of latent variables $\mathbf{C}_{1:t} = \{i_{1:t}, C_{1:t}, S_{1:t}\}$. The joint posterior distribution can be described as follows:

$$\begin{aligned} & p(x_{0:t}, \mathbf{C}_{1:t}, LM, \Theta, m \mid u_{1:t}, z_{1:t}, y_{1:t}, f_{1:t}, AM, \mathbf{h}) \\ &= p(LM \mid S_{1:t}, \lambda) p(\Theta \mid x_{0:t}, \mathbf{C}_{1:t}, f_{1:t}, \mathbf{h}) p(m \mid x_{0:t}, z_{1:t}) \\ &\quad \cdot \underbrace{p(x_{0:t}, \mathbf{C}_{1:t} \mid u_{1:t}, z_{1:t}, y_{1:t}, f_{1:t}, AM, \mathbf{h})}_{\text{Particle filter}} \end{aligned} \tag{S17}$$

where the set of hyperparameters is denoted by $\mathbf{h} = \{\alpha, \beta, \gamma, \chi, \lambda, m_0, \kappa_0, V_0, \nu_0\}$.

The variables of the joint posterior distribution can be learned by Gibbs sampling, which is a Markov chain Monte-Carlo-based batch learning algorithm, in a manner similar to the nonparametric Bayesian spatial concept acquisition method (SpCoA) (Taniguchi et al., 2016). In addition, learning can be realized in a spatial concept formation model after the map is generated via any other SLAM.

The learning procedure of SpCoSLAM for each step is described as follows:

(a) The robot obtains weighted finite-state transducer (WFST) speech recognition results $\mathcal{L}_{1:t}$ from the user speech signals $y_{1:t}$ using a language model $LM$. The WFST is a word graph, i.e., a lattice format, which alternatively represents the $N$-best speech recognition results. Initially, a phoneme dictionary is provided as the language model $LM$ without a prior word list.
(b) The WFST speech recognition results $\mathcal{L}_{1:t}$ are segmented to the word sequences $S_{1:t}$ using an unsupervised word segmentation approach referred to as latticelm (Neubig et al., 2012).
(c) The latent variable $x_t$ and importance weight $\omega_z$ regarding self-localization are obtained by the grid-based FastSLAM 2.0 from control data $u_t$, depth data $z_t$ and particles that represent the self-positions $x_{t-1}$ at the previous time step.
(d) The latent variables $i_t, C_t$ of spatial concepts are sampled by the proposal distribution on the particle filter.
(e) The importance weights $\omega_s, \omega_f$ are obtained as the marginal likelihoods of observations $S_t, f_t$.
(f) The environmental map $m$ is updated by self-positions $x_{0:t}$ and depth data $z_{1:t}$.
(g) The set of model parameters $\Theta$ of the spatial concepts are estimated from the observation $f_{1:t}$ and sampled variables $x_{0:t}, \mathbf{C}_{1:t}$.

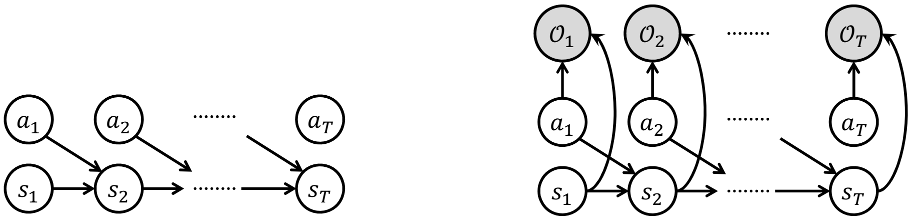


**Figure S1.** Left: Graphical model of Markov decision process (MDP) with states and actions. Right: Graphical model for CaI with the optimality variables. This additional variable is a binary random variable, where $\mathcal{O}_t = 1$ denotes that the time-step $t$ is optimal.

(h) The language model $LM$ is updated by adding words $S^*_{1:t}$ in a particle of maximum weight to the initial dictionary.

(i) The particles are re-sampled according to their weights $\omega_t = \omega_z \cdot \omega_s \cdot \omega_f$.

Steps (b) – (g) are performed for each particle. See the original paper (Taniguchi et al., 2017) for details.

## 3 CONTROL AS PROBABILISTIC INFERENCE

The theoretical gap between the control problems, including reinforcement learning (RL) and the probabilistic inference in the generative model, was bridged by CaI (Levine, 2018).

In general decision-making problems, including RL, a policy that maximizes the expected cumulative reward is estimated as follows:

$$\vartheta^\star = \underset{\vartheta}{\operatorname{argmax}} \sum_{t=1}^{T} \mathbb{E}_{(s_t,a_t)\sim p(s_t,a_t|\vartheta)} \left[ r(s_t, a_t) \right], \tag{S18}$$

where $r(s_t, a_t)$ is a reward function, $s_t$ is a state variable, $a_t$ is an action variable, $\vartheta$ is a parameter for the policy function, and $\vartheta^*$ is an optimal policy parameter. It should be noted that $s_t$ and $a_t$ correspond to $x_t$ and $u_t$ in our method, respectively.

With respect to CaI, the planning problem can be formulated as an inference from the probabilistic graphical model. Figure S1 presents the graphical models of the Markov decision process (MDP) with an optimality variable $\mathcal{O}_t$. In the graphical model for CaI, the distribution denoting the generative process on the binary random variable $\mathcal{O}_t$ is represented as

$$p(\mathcal{O}_t = 1 \mid s_t, a_t) = \exp(r(s_t, a_t)). \tag{S19}$$

The maximum a posteriori inference in the posterior distribution $p(\tau \mid o_{1:T})$ corresponds to a type of planning problem. Here, trajectory is $\tau = \{s_{1:T}, a_{1:T}\}$ and the set of optimality variables is $o_{1:T} = \{\mathcal{O}_t =$

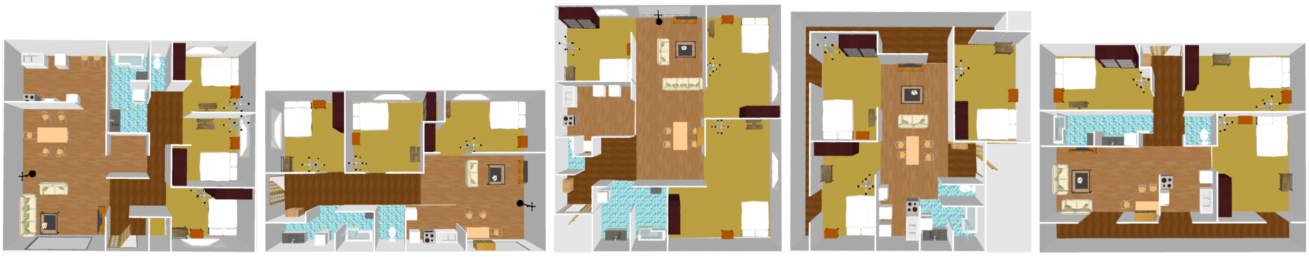

**Figure S2.** Home environments created with SIGVerse in Experiment I

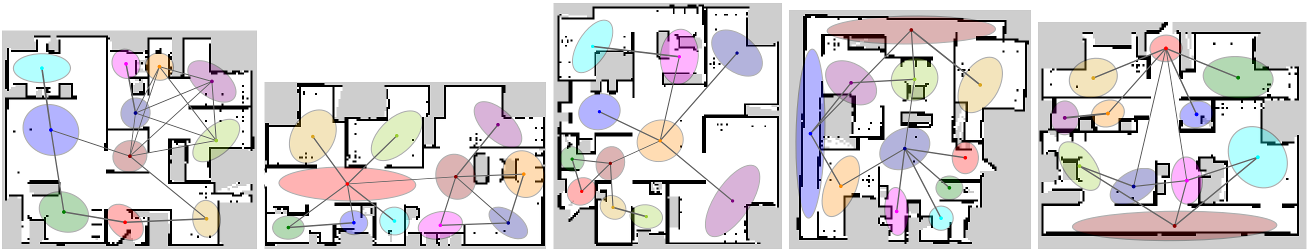

**Figure S3.** Spatial concepts on home environments in Experiment I

$1\}_{t=1}^{T}$. The posterior distribution over actions when we condition $\mathcal{O}_t = 1$ for all $t \in \{1, \ldots, T\}$ is shown as

$$p(\tau \mid o_{1:T}) \propto p(s_1) \prod_{t=1}^{T} p(s_{t+1} \mid s_t, a_t) p(\mathcal{O}_t = 1 \mid s_t, a_t)$$
$$= \underbrace{\left[ p(s_1) \prod_{t=1}^{T} p(s_{t+1} \mid s_t, a_t) \right]}_{\text{State-transition with action}} \exp \underbrace{\left( \sum_{t=1}^{T} r(s_t, a_t) \right)}_{\text{Cumulative reward}}. \quad \text{(S20)}$$

This suggests that the optimalities $o_{1:T}$ are given as observations in a HMM-style model. Therefore, the trajectory probability is given by the product between its probability to occur according to the dynamics and the exponential of the cumulative reward along that trajectory.

In this case, the policy function can be expressed as $\pi_\vartheta(s_t, a_t) = p(a_t \mid s_t, \vartheta)$. The optimal policy function can be expressed as $p(a_t \mid s_t, \vartheta^\star) \approx p(a_t \mid s_t, o_{t:T})$; then, the right side is not related to the parameter $\vartheta$.

Levine (2018) has described that the CaI allows for the application of various techniques of probabilistic inference, e.g., the forward–backward algorithm and variational inference, for control and planning problems.

## 4 SIMULATOR ENVIRONMENTS AND THEIR SPATIAL CONCEPTS IN EXPERIMENT I

This section introduces the simulated home environments generated in SIGVerse and outlines the spatial concepts explored in Experiment I. Figure S2 shows the home environments. Figure S3 shows It includes eleven spatial concepts and position distributions for each environment. The terminology associated with each place varies depending on the environment. The list of words for spatial concepts is provided

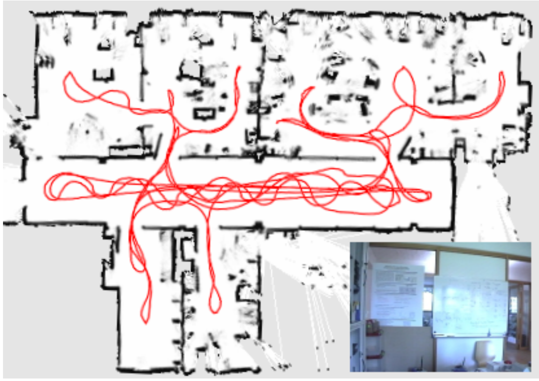

**Figure S4.** The robot trajectory within the dataset for learning is depicted by a red curved line, while an example image captured by the robot is shown in Experiment II. This image is included in the dataset (Stachniss, 2003).

below: "Entrance", "Living room", "Dining room", "Kitchen", "Bathroom", "Dressing room", "Lavatory", "Bedroom", "Storage space", "Corridor", "Open space", "East side", and "South side"

## 5 DATASET OF ALBERT-B-LASER-VISION IN EXPERIMENT II

The experimental environment matched that of the open dataset albert-b-laser-vision, sourced from the robotics dataset repository (Radish) (Stachniss, 2003). This dataset was captured using the b21r robot Albert within Building 79, University of Freiburg. The dataset comprises a log file containing odometry, laser range data, and image data. Detailed information about the robot's properties is provided within the dataset. Figure S4 shows the robot trajectory image of the dataset.

## REFERENCES


Doucet, A., De Freitas, N., Murphy, K., and Russell, S. (2000). Rao-Blackwellised particle filtering for dynamic Bayesian networks. In *Proceedings of the 16th Conference on Uncertainty in artificial intelligence* (Morgan Kaufmann Publishers Inc.), 176–183. doi:10.1007/978-1-4757-3437-9_24

Gildea, D. and Hofmann, T. (1999). Topic-based Language Models Using EM. In *Proceedings of the European Conference on Speech Communication and Technology (EUROSPEECH)*

Grisetti, G., Stachniss, C., and Burgard, W. (2007). Improved Techniques for Grid Mapping with Rao-Blackwellized Particle Filters. *IEEE Transactions on Robotics* 23, 34–46

Levine, S. (2018). Reinforcement Learning and Control as Probabilistic Inference: Tutorial and Review. *arXiv preprint arXiv:1805.00909*

Montemerlo, M., Thrun, S., Koller, D., Wegbreit, B., and Others (2003). FastSLAM 2.0: An improved particle filtering algorithm for simultaneous localization and mapping that provably converges. In *Proceedings of the International Joint Conference on Artificial Intelligence (IJCAI)* (Acapulco, Mexico), 1151–1156

Murphy, K. P. (2012). *Machine learning: a probabilistic perspective* (Cambridge, MA: MIT Press)

Neubig, G., Mimura, M., Mori, S., and Kawahara, T. (2012). Bayesian learning of a language model from continuous speech. *IEICE Transactions on Information and Systems* 95, 614–625

[Dataset] Stachniss, C. (2003). The Robotics Data Set Repository (Radish)

Taniguchi, A., Hagiwara, Y., Taniguchi, T., and Inamura, T. (2017). Online Spatial Concept and Lexical Acquisition with Simultaneous Localization and Mapping. In *Proceedings of the IEEE/RSJ International Conference on Intelligent Robots and Systems (IROS)*. 811–818. doi:10.1109/IROS.2017.8202243

Taniguchi, A., Taniguchi, T., and Inamura, T. (2016). Spatial Concept Acquisition for a Mobile Robot that Integrates Self-Localization and Unsupervised Word Discovery from Spoken Sentences. *IEEE Transactions on Cognitive and Developmental Systems* 8, 285–297. doi:10.1109/TCDS.2016.2565542
